# Simultaneous Determination of Fluoroquinolones and Sulfonamides Originating from Sewage Sludge Compost

**DOI:** 10.1155/2017/9254072

**Published:** 2017-06-12

**Authors:** K. Kipper, M. Lillenberg, K. Herodes, L. Nei, E. Haiba

**Affiliations:** ^1^Institute of Chemistry, University of Tartu, Tartu, Estonia; ^2^Estonian University of Life Sciences, Tartu, Estonia; ^3^Tartu College, Tallinn University of Technology, Tartu, Estonia

## Abstract

A simultaneous method for quantitative determination of traces of fluoroquinolones (FQs) and sulfonamides (SAs) in edible plants fertilized with sewage sludge was developed. The compounds were extracted from the plants by rapid and simple liquid extraction followed by extracts clean-up using solid phase extraction. The eluent additive 1,1,1,3,3,3-hexafluoro-2-propanol was used for liquid chromatographic detection to achieve separation of structurally similar antimicrobials like ciprofloxacin and norfloxacin. Identification and quantification of the compounds were performed using high-performance liquid chromatography with electrospray ionization mass spectrometry in selected reaction monitoring mode. Method was validated and extraction recoveries of FQs and SAs ranged from 66% to 93%. The limit of quantifications was from 5 ng/g in the case of ofloxacin to 40 ng/g for norfloxacin. The method precision ranged from 1.43% to 2.61%. The developed novel method was used to evaluate the plats antimicrobial uptake (potato* (Solanum tuberosum* L*.)*, carrot* (Daucus carota* L*.)*, lettuce* (Lactuca sativa* L*.)*, and wheat* (Triticum vulgare* L*.)*) from soil and migration of the analytes inside the plants.

## 1. Introduction

The increase of the yearly production of sewage sludge compost containing human and veterinary antimicrobials has led to antimicrobial resistance being one of the top health challenges in the 21st century [1]. One of the largest and most diverse microbial habitats on Earth is soil, a vast repository of the antimicrobial resistance genes between soil bacteria and clinical pathogens [2].

When antimicrobials are eliminated from the human body, they can be excreted in their native form or as metabolites [3]. Since antimicrobials are developed to have a specific mode of action, even low levels of these drugs in edible plants can cause effects in organisms [4].

Several studies have demonstrated that the two most important sources through which toxic compounds reach the environment are sewage sludge and compost, which are often used in agriculture [5–9]. More generally, pharmaceuticals move into the sewage system and to waste water treatment plants [10]. The nutrition-rich sewage sludge and compost can be used as fertilizers for plants. The increasing proportions of administered drugs and personal care products are alarming because the compound releases into the environment are not controlled [11, 12] and this is a potential threat to the environment [13–15]. It is worrisome that pharmaceutical compounds may potentially enter edible food plants that have been fertilized with sewage sludge compost [9, 16–18].

The risks from the fertilizer should be evaluated carefully. Exposure to pharmaceuticals via plant-derived foodstuffs is usually low and effects on human health are in most cases unlikely. This route of exposure may, however, be more significant for a small number of highly toxic medicines or in situations where long-term low-level exposure could elicit subtler effects (e.g., promotion of antibacterial resistance or endocrine disruption) [19]. A chemical can undergo various structural changes by a multitude of biotic and nonbiotic processes after its introduction into the environment. Structural transformations may also be a result of effluent treatment [4, 20–26]. The maximum residue levels (MRL) are set only for food of animal origin, milk and meat [27, 28].

Analytical methods have been developed and applied for the determination of different antimicrobials in sewage sludge and its compost, biosolids, and sludge-treated soil [29–38]. Many antimicrobials, known to be persistent in soils fertilized with sewage sludge compost, can accumulate into food plants [39–46]. The pharmaceuticals accumulated in the food plants may generate resistant bacteria in human and animal organisms. The groups of antimicrobials of interest are well-known, but it is a complicated task to separate structurally similar compounds in a reversed-phase LC (liquid chromatography) system. On the other hand, the concentrations in residue levels are very low and therefore improved MS (mass spectrometry) sensitivity is more than welcome. The aim of the present study was to use an eluent additive 1,1,1,3,3,3-hexafluoroisopropanol (HFIP) to improve LC separation significantly with alternative selectivity in C18 stationary phase and enhance MS detection of fluoroquinolones (FQs) and sulfonamides (SAs) in small concentration levels to quantify them in food plant samples. These drugs were selected according to three criteria: (1) their stability in soil [47], (2) their potential to accumulate into plants [39, 46], and (3) their presence in sewage sludge and its compost [48].

For the prevention of the development of microbial resistance of humans and animals, the concentration of antimicrobials in compost must be significantly below 1 *μ*g/kg, securing the relevant soil concentrations at 0.01–0.1 *μ*g/kg [49]. In our previous work [16], the highest detected concentrations of the antimicrobial norfloxacin (NOR), ciprofloxacin (CIP), ofloxacin (OFL), sulfamethoxazole (SMX), and sulfadimethoxine (SDM) in sewage sludge and its compost were as shown in Table 1.

## 2. Materials and Methods

### 2.1. Chemicals

Pharmaceuticals were purchased from Riedel-de-Haën (Seelze, Germany): three FQs, CIP (purity 99.8%), NOR (purity 99.9%), and OFL (purity 99.3%); two SAs, SDM (purity 99.4%) and SMX (purity 99.9%). Acetonitrile and methanol were obtained from J.T. Baker (Deventer, Netherlands), HPLC grade formic acid, and ammonia from Riedel-de-Haën. HFIP was purchased from Sigma (St. Louis, MO, USA). All solvents were of reagent grade or higher quality. Water was purified (18.2 MΩ  × cm at 25°C and a TOC value below 3 ppb) in-house using a Milli-Q Plus system from Millipore (Bedford, USA). Hydrophilic-lipophilic balanced (HLB) solid phase extraction (SPE) cartridges (Oasis HLB (60 *μ*m), 500 mg/6 mL) were purchased from Waters (Milford, MA, USA).

The selection of antimicrobials was made according to preliminary pilot study on antimicrobials usage and presence in the sewage sludge samples [48], their stability in the soil and potential degradation during the composting procedure [16], and their uptake from the soil by plants [46].

### 2.2. Plant Samples

For the experiments with plants, the antimicrobials were spiked into the soil in which the plants were grown [49]. Aqueous solutions of the studied pharmaceuticals were mixed with soil. The final concentration of each pharmaceutical was 10 mg per kg of dry soil. To ensure better dissolution of the studied pharmaceuticals, fluoroquinolones were dissolved in 2 ml of 0.1 mM ammonium acetate buffer solution with pH 2.8 and sulfonamides were dissolved in 2 ml of 0.3 M NaOH. Potatoes* (Solanum tuberosum* L.), carrots* (Daucus carota* L.), lettuce* (Lactuca sativa* L.), and wheat* (Triticum vulgare* L*.)* were grown in the presence of five antimicrobials commonly present in sewage sludge (CIP, NOR, OFL, SDM, and SMX). The potato tubers and plant seeds were planted into the pots, with one tuber or 35 seeds in each pot. The plants were cultivated in a greenhouse under natural light conditions for 120 days after planting (lettuce, 70 days). The soil was weighed, and aqueous solutions of the studied pharmaceuticals were mixed with the soil. The final concentration of each pharmaceutical was 0.01, 0.1, 0.5, 1, and 10 mg/kg (dry weight). Three parallel experiments were conducted for each concentration of antimicrobials. In reference experiments, plants were cultivated in antimicrobial-free soils. The plants were collected and washed carefully. Potatoes and carrots were chopped into ca 1 cm^3^ pieces. Then the plants were dried at room temperature in the darkness and after that milled for analyses, using Knifetec 1095 Sample Mill (Foss) and a common coffee mill. The size of the particles of the powder was <1 mm^3^. The milled samples were dewatered in a thermostat at 45°C for 24 hours using thermostat Binder KB 115 and stored in hermetic plastic bags for three weeks at −80°C (using refrigerator Sanyo MDF-U54V) before analysis.

### 2.3. Sample Preparation

250 mg of dried plants (grains, roots, or leaves) was extracted with 10 mL of a 1 : 1 (v/v) mixture of acetonitrile and 1% acetic acid, then homogenized with laboratory homogenizer DIAX 900 (Heidolph Instruments, Germany) at 25,000 rpm, sonicated (5′), vortexed (1′), and centrifuged at 8000 rpm. The supernatant was then separated and dried by nitrogen stream to remove acetonitrile. Approximately 15 mL of 1% acetic acid was added to the 1 mL of evaporation residue [49].

The extract collected with liquid extraction was cleaned up with solid phase extraction (SPE). Antimicrobials, CIP, NOR, OFL, SDM, and SMX, were extracted using HLB cartridges. For the SPE procedure, the vacuum manifold (Agilent Technologies) was used. HLB cartridges were preconditioned with 20 mL of methanol and 10 mL of Milli-Q water. The sample was loaded at a rate of 6 mL/min. After extraction, the compounds were eluted from the cartridges using 12 mL of methanol. The SPE extracts were evaporated to dryness in polypropylene vials in an N2 stream. Residue was dissolved in 1 mL of 20% methanol with buffer solution (5 mM 1,1,1,3,3,3-hexafluoro-2-propanol, pH adjusted to 9.0 with NH_4_OH).

### 2.4. Liquid Chromatography-Mass Spectrometry

Chromatographic separation of the analytes was carried out on the Agilent Series 1100 LC-MSD Trap XCT (Agilent Technologies, Santa-Clara, CA, USA) equipped with a binary pump, a degasser, an autosampler, and a column thermostat. Five antimicrobials were chromatographed using a Waters XBridge C18 column (150 mm × 3 mm, 3.5 *μ*m) equipped with a Waters Guard Cartridge (20 mm × 4.6 mm) (Waters, Milford, USA). For detection, a diode array detector and ESI-MS were used in series. ESI-MS detection was carried out in positive ion detection mode. Selected reaction monitoring was used. Full MS^2^ spectra were recorded and the following transitions were applied for quantification: OFL *m*/*z* 362→261, 318; NOR *m*/*z* 320→302, 276; CIP *m*/*z* 332→288, 314; SMX *m*/*z* 254→108, 188; SDM *m*/*z* 311→108, 156, 218, 245. Default parameters for ESI and MS were used for all the experiments (nebulizer gas pressure was 40 psi, dry gas flow was 10 L/min, dry gas temperature was 350°C, capillary voltage was 5000 V, detected mass range was from *m*/*z* 100 to 1000, and target mass for compounds was *m*/*z* 350). The LC-MS instrument was controlled by Agilent Chemstation for LC 3D rev. A.10.02 (Agilent Technologies) and LC/MSD Trap Control ver. 5.2 (Bruker Daltonik GmbH, Germany). Data analysis was carried out using Chemstation software (Agilent Technologies) and Data Analysis for LC/MSD Trap Version 3.2 (Bruker Daltonik GmbH).

### 2.5. Chromatographic Conditions

5 mM HFIP buffer (pH adjusted with NH_4_OH to 9.0) and methanol were used for elution. Gradient elution at flow rate 0.3 mL/min started at 10% methanol and was raised to 55% within 25 min, after which methanol concentration was raised to 100% within 5 min. Methanol concentration was kept at 100% for 5 min, then lowered to 10% in 5 min, and equilibrated at 10% for 5 min. Column temperature was set to 30°C and the injection volume was 10 *μ*L.

### 2.6. Standard and Buffer Solutions

Stock solutions of the analytes at 1 mg/mL in the appropriate solvent (mixture of MeOH and 1 mM ammonium acetate buffer with 0.1% formic acid, 20/80) were prepared. The stock solution for SDM was 0.5 mg/mL due to its poor solubility. The working standard solution contained 5 antimicrobials at 0.1 mg/mL. From this solution dilution (10 *μ*g/mL and 1 *μ*g/mL) was made. The stock solution was stored at −20°C. Fresh working standard solutions were prepared daily.

### 2.7. Method Validation

The developed method was validated following Eurachem guidelines [50] and the linearity, limit of quantification, and process efficiency (recovery and matrix effect) were evaluated.

## 3. Results and Discussion

### 3.1. Liquid Extraction

The developed method is based on the combination of liquid extraction, SPE, and LC-MS analysis of a total of five antimicrobials. Antimicrobials from two classes, FQs and SAs, are structurally and chemically diverse. The variables optimized were extraction solvent, pH, and homogenization. Hexane, chloroform, methanol, and acetonitrile were tested as extraction solvents. The organic solvent content in an extraction solvent was varied from 20 to 100%. Extraction with chloroform and hexane gave the lowest overall antibiotic recoveries (1-2%) for CIP and NOR. The extraction mixture's aqueous solution's pH varied from acidic (1% acetic acid, pH 2.0) to basic (5 mM ammonium acetate, pH 9.0 (using NH_4_OH)) conditions. Extraction with acetonitrile was more efficient compared with methanol. The mixture of acetonitrile and 1% acetic acid (1/1) was finally chosen as an extraction solvent for simultaneous extraction of all the analytes of interest. During the optimization of liquid extraction, it was found that extraction efficiency increased when homogenization was used along with sonication and mixing. The increase of the time of liquid extraction stages did not increase extraction recoveries. In total, the time for a LE procedure was 17 minutes.

### 3.2. Solid Phase Extraction (SPE)

After liquid extraction and centrifugation, the supernatant was separated and dried by nitrogen stream to remove acetonitrile. Remaining extracts were cleaned up with HLB SPE cartridges. The HLB cartridges enable retaining both hydrophilic and hydrophobic compounds [51] and give the highest recoveries for all of the analytes studied. The sample pH was adjusted by adding approximately 15 mL of 1% acetic acid. For elution, methanol was used.

### 3.3. LC-MS

**Figure 1:**
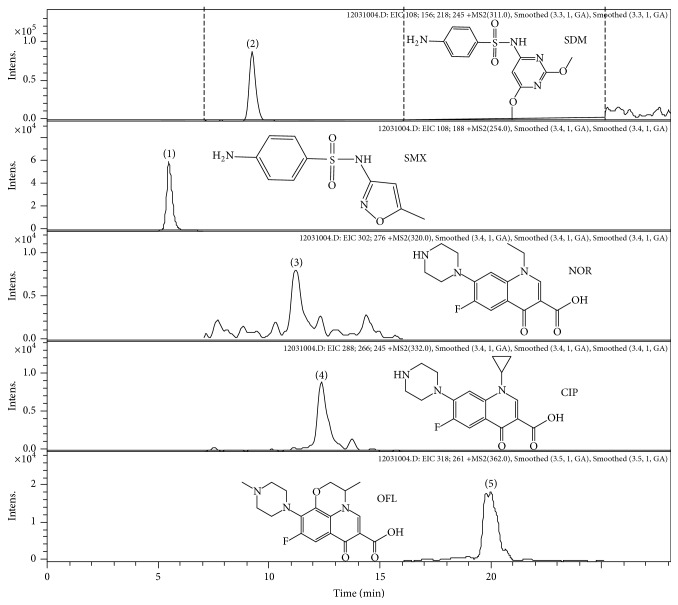
The chromatographic separation of antibiotic residues in potato tuber. Antimicrobials spiked in the LoQ level ((1) SMX 32.7 ng/g; (2) SDM 17.8 ng/g; (3) NOR 40.0 ng/g; (4) CIP 27.2 ng/g; (5) OFL 5.0 ng/g). Eluent: 5 mM HFIP (pH 9) and MeOH. Analytical column: Waters XBridge C18 column (150 mm × 3 mm, 3.5 *μ*m).

Previously several buffer solutions and pH values were tested thoroughly for the reverse phase (RP) LC separation of named antibiotics [31]. Although the MS signal of analytes was higher using an eluent with a lower pH (ammonium acetate, formic acid with pH 2.8), the separation of the compound was not followed. At higher pH values, the fluoroalcohol HFIP gave significantly better ionization efficiencies and better peak shapes, compared to acidic conditions and known buffer additives [47], and all 5 compounds had baseline separation for an antibiotic standard solution using RP column. For the LC analysis, the 5 mM HFIP and gradient elution with methanol was used. The application demonstrates the successful separation of chosen compounds from the potato tubers extract (Figure 1).

HFIP as an eluent additive is predominantly protonated at pH 9.0. Therefore, the interaction with the nonpolar stationary phase is relatively strong [31, 52]. HFIP covers the C18 stationary phase with a fluorous layer [52], shifting the stationary phase properties nearer to a fluorinated stationary phase. Acting as a weak ion-pairing agent, HFIP allows the alternative interaction with structurally similar FQs [52]. Using HFIP as an eluent additive decreases the retention of SAs more than FQs. At pH 9.0, the FQs exist mostly in the zwitterionic forms. Therefore, the retention pattern of FQs is influenced both by the fluorous layer of the stationary phase and by the acid-base equilibrium. At the same time, the SAs pKa values are much lower than 9 and the mobile phase pH does not affect the retention changes strongly.

**Figure 2:**
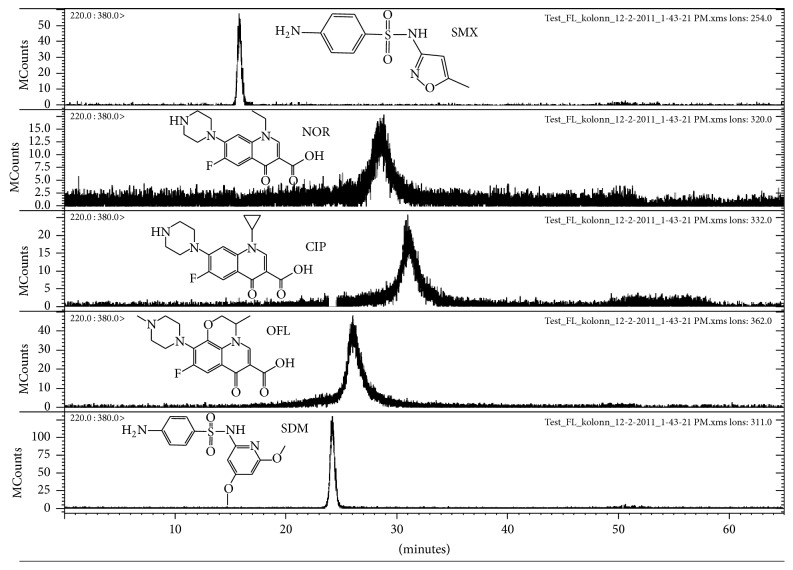
MS chromatogram for the standard solution of FQs and SAs (10 *μ*g/g). Eluent: (ammonium acetate, formic acid pH 2.8) and MeOH. Used analytical column: Epic FO-LB C8 column (150 mm × 3 mm, 3.5 *μ*m).

In order to attain higher selectivity for FQs, the separation of five antimicrobials was studied in the alkyl perfluorinated stationary phase Epic FO-LB C8. However, the SMX and SDM showed well-expressed peak shapes and had different retention; the FQs peaks were wide and shallow, having similar retention in the perfluorinated stationary phase (Figure 2). Fluorinated analytes retention on the fluorinated stationary phase is mainly influenced by the number of fluorine atoms in the analyte molecule [53]. The number of fluorine atoms in the three FQs studied is one and the structures of the molecules are similar. Therefore, the retention of the analytes on the fluorinated stationary phase is also similar. On the other hand, the ESI signal of the analytes should be enhanced under acidic conditions. The MS chromatogram of the FQs and SAs separation had a high noise level in the extracted ion chromatograms for FQs; the peaks were broad and partly overlapping (Figure 2). Neither better separation nor enhanced signal was obtained by optimization of the elution gradient or the buffer composition or pH.

### 3.4. Method Validation

**Figure 3:**
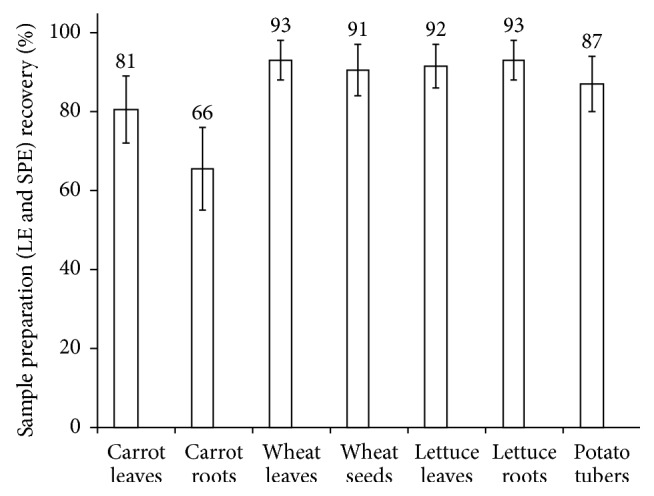
Average sample preparation (LE and SPE) recoveries (*n* = 2) of 5 antimicrobials (CIP, ciprofloxacin; NOR, norfloxacin; OFL, ofloxacin; SDM, sulfadimethoxine; SMX, sulfamethoxazole) from different parts of food plants grown in loamy soil using LE and SPE. Error bars show the recovery ranges.

The described method was validated for the simultaneous determination of CIP, NOR, OFL, SDM, and SMX in plants. For calibration, antimicrobials and standard solutions were prepared in 10% methanol and water. The calibration graphs with peak area versus concentration were composed in concentration range 5–10,000 ng/g and were linear with *r*^2^ > 0.9998. Extraction recovery was calculated from standard addition experiments. Extraction recoveries for all detected pharmaceuticals in all matrices varied from 54 to 98%; the average recoveries are shown in Figure 3. Method validation was performed in the matrix which showed the lowest recovery: carrot roots in loamy soil (recovery ranges 54–78%, average recovery 66%). A postextraction spike in three different concentrations over the calibration range (low, 5 ng/g; medium, 250 ng/g; and high, 5,000 ng/g) to the different plants did not show a significant matrix interference. The process efficiency was primarily influenced by the extraction recovery in the two steps (LLE and SPE) of the sample preparation.

**Figure 4:**
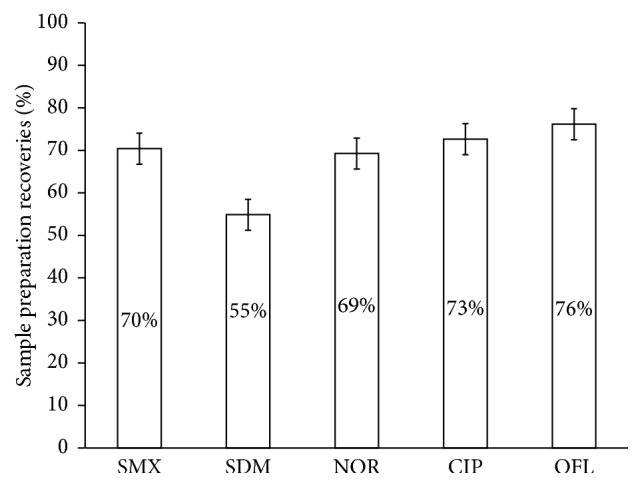
Average sample preparation recoveries for 5 antimicrobials from carrot roots using LE and SPE. Matrix: carrot roots. Error bars are 2 times standard deviation. CIP, ciprofloxacin; NOR, norfloxacin; OFL, ofloxacin; SDM, sulfadimethoxine; SMX, sulfamethoxazole.

The average recoveries of antimicrobials from carrot roots, as shown in Figure 4, were 73% (CIP), 69% (NOR), 76% (OFL), 55% (SDM), and 70% (SMX). Standard deviations for the recoveries were 1% (CIP), 2% (NOR), 2% (OFL), 1% (SDM), and 1% (SMX). The limits of quantifications (LoQs) were estimated as ten times the standard deviation from five replicate analyses of unspiked and spiked plant samples using HLB cartridges. LoQs were as follows: CIP 27.2; NOR 40.0; OFL 5.0; SDM 17.8; and SMX 32.7 ng/g. The relative standard deviations (RSD) were, respectively, 0.27, 0.40, 0.05, 0.18, and 0.32 percent.

## 4. Conclusion

Plant uptake of pharmaceutical residues, present (even in very small amounts) in soils fertilized with sewage sludge compost, is an obvious reality. As antimicrobials consumed in very small amounts with everyday food can initiate strains of resistant bacteria in human and animal organisms, the high sensitivity of their detection methodology is of utmost importance. Improved separation of the groups of structurally similar antimicrobials, fluoroquinolones (FQs) and sulfonamides (SAs), and enhanced MS signal intensities were achieved as a result of this work, by using an eluent additive HFIP in regular C18 stationary phase. The developed and validated method described in the current paper has turned out to be an efficient tool for detecting the concentrations of antimicrobials in food plants fertilized with sewage sludge compost.

## Figures and Tables

**Table 1:** Occurrence of antimicrobials (*μ*g/kg) in sewage sludge and its compost (illustrative data).

Antimicrobial media	NOR	CIP	OFL	SMX	SDM
Sewage sludge	162	426	39	6	20
Compost	22	20	3	1	4
